# Estimated 8-year cumulative incidence of diabetes mellitus among Sami and non-Sami inhabitants of Northern Norway - The SAMINOR Study

**DOI:** 10.1186/s12902-019-0399-7

**Published:** 2019-06-24

**Authors:** Ali Naseribafrouei, Bent Martin Eliassen, Marita Melhus, Johan Svartberg, Ann Ragnhild Broderstad

**Affiliations:** 1 Centre for Sami Health Research, Department of Community Medicine, Faculty of Health Sciences, UiT The Arctic University of Norway, Box 6050 Langnes, N-9037 Tromsø, Norway; 2 Faculty of Nursing and Health Sciences, Nord University, Bodø, Norway; 3 Division of Internal Medicine, University Hospital of North Norway, Tromsø, Norway; 4 Tromsø Endocrine Research Group, Department of Clinical Medicine, UiT The Arctic University of Norway, Tromsø, Norway; 5 Department of Medicine, University Hospital of North Norway, Harstad, Norway

**Keywords:** Cumulative incidence, Diabetes mellitus, Indigenous, Native, Norwegian, SAMINOR, HbA1c, Sami

## Abstract

**Background:**

The aim of the study was to estimate and compare the 8-year cumulative incidence of diabetes mellitus (DM) among Sami and non-Sami inhabitants of rural districts in Northern Norway.

**Methods:**

Longitudinal study based on linkage of two cross-sectional surveys, the SAMINOR 1 Survey (2003–2004) and the SAMINOR 2 Clinical Survey (2012–2014). Ten municipalities in rural Northern Norway were included in the study. DM-free participants aged 30 and 36–71 years in SAMINOR 1 were followed from 2 years after SAMINOR 1 to attendance in SAMINOR 2. The average follow-up time was 8.1 years. Of 5875 subjects who had participated in SAMINOR 1 and could potentially be followed to SAMINOR 2, 3303 were included in the final analysis. Self-reported DM and/or HbA1c ≥ 6.5% were used to identify incident cases of DM.

**Results:**

At baseline, body mass index (BMI) and waist-to-height ratio (WHtR) were higher among Sami than among their non-Sami counterparts. After 8 years of follow-up, 201 incident cases of DM were identified (6.1% both Sami and non-Sami subjects). No statistically significant difference was observed in the cumulative incidence of DM between the Sami and non-Sami.

**Conclusions:**

No statistically significant difference in the 8-year cumulative incidence of DM among Sami and non-Sami was observed, although Sami men and women had higher baseline BMI and WHtR.

## Background

Type 2 diabetes mellitus (DM) is one of the most prevalent and disabling chronic diseases affecting millions of people worldwide [1]. Indigenous peoples throughout the world are facing an unprecedented epidemic of type 2 DM [2], but publications concerning the incidence of the disease among these groups are rather sparse. This could in part be due to the need for costly and cumbersome cohort studies or the lack of available robust data from national registries.

The Sami are an indigenous people, who for centuries have inhabited northern parts of Norway, Sweden, and Finland, and the Kola Peninsula of Russia. Sami people might possess genes that either predispose them to or protect them against development of diseases like DM. Furthermore, they have their own culture, diet, and so forth, which might play a role in increasing or decreasing the risk of DM. Internationally, studies have shown a striking difference in the prevalence and incidence of diabetes mellitus between indigenous populations and majority populations [3–6]. Higher incidence and prevalence of type 2 DM among indigenous peoples, in comparison to the benchmark populations, seems to be a shared phenomenon worldwide [2]. For example, the age-standardised incidence of type 2 DM of 1814 Australian Aboriginal and Torres Strait Islander adults from 1999 to 2007 was reported to be 30.5 in 1000 person-years. This incidence rate is nearly four times higher than that for the non-Indigenous population and 50% higher than the incidence reported 10 years ago in Australian Aboriginals [7].

Previous research based on data from 24 municipalities in the SAMINOR 1 Survey (2003–2004), showed no statistically significant difference between Sami and non-Sami in the prevalence of DM, defined by self-report and/or non-fasting plasma glucose ≥11.1 mmol/L [8, 9]. However, a study using data from the SAMINOR 2 Clinical Survey (2012–2014), found higher prevalence of both pre-diabetes and type 2 DM among Sami people, when self-report and/or HbA1c ≥ 6.5% was used to define diabetes cases [10]. However, due to the different population samples and diagnostic methods applied, it is not possible to ascertain whether the higher diabetes prevalence in SAMINOR 2 among Sami participants reflects a higher incidence of diabetes over the last decade. A study from the SAMINOR 1 Survey, showed higher obesity prevalence and a more sedentary lifestyle among Sami women [11]. Therefore, they are expected to have higher risk of developing type 2 DM.

To our knowledge, there are no previous studies investigating the incidence of DM in the Sami population of rural municipalities in Northern Norway.

The aim of this study is to measure and compare the 8-year cumulative incidence of DM among Sami and non-Sami inhabitants of rural districts in Northern Norway.

## Methods

In 2003–2004, the Centre for Sami Health Research at UiT The Arctic University of Norway, in collaboration with the Norwegian Institute of Public Health, conducted the SAMINOR 1 Survey (hereafter referred to as SAMINOR 1) [12]. This survey included 24 mostly rural municipalities and districts in Northern and Central Norway with a considerable proportion of Sami inhabitants.

**Fig. 1:**
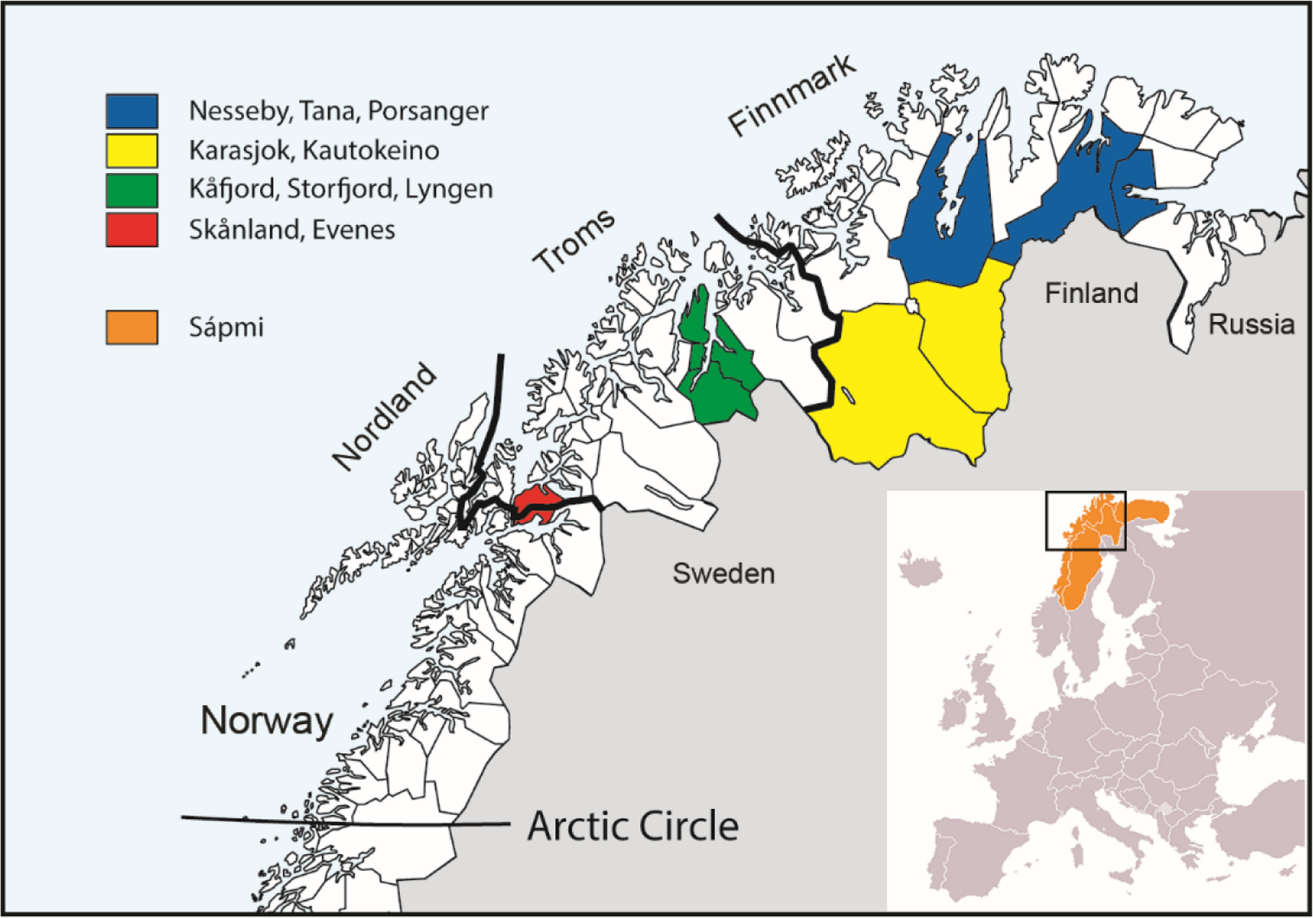
Map of Northern Norway, Sápmi, and the included municipalities in the SAMINOR 2 Clinical Survey (2012–2014). Published with permission from Centre for Sami Health Research

In 2012–2014, the Centre for Sami Health Research undertook a two-part second survey, the SAMINOR 2 Questionnaire Survey [13] and the SAMINOR 2 Clinical Survey [14]. The present analyses are based on data from the SAMINOR 2 Clinical Survey (hereafter referred to as SAMINOR 2), which, similarly to SAMINOR 1, consisted of self-administered questionnaires, a clinical examination, and analysis of blood samples. The survey was conducted in 10 rural municipalities in Finnmark, Troms, and Nordland counties, all previously included in SAMINOR 1: Kautokeino, Karasjok, Tana, Nesseby, Porsanger, Lyngen, Storfjord, Kåfjord, Skånland, and Evenes (Fig. 1). The survey was conducted in 10 rural municipalities in Finnmark, Troms, and Nordland counties, all previously included in SAMINOR 1: Kautokeino, Karasjok, Tana, Nesseby, Porsanger, Lyngen, Storfjord, Kåfjord, Skånland, and Evenes (Fig. 1).

The included municipalities were chosen due to a high number of Sami inhabitants. The invitees were informed that the study aimed primarily to increase knowledge about health, diseases, and living conditions in regions with Sami and Norwegian populations and provide a health profile for their county/municipality, in addition to getting personal feedback of their own health status.

### Study sample

In SAMINOR 1, a total of 27,987 subjects, aged 30 or 36–79 years were invited, and 16,865 participated (60.6%). In SAMINOR 2, 12,455 subjects, aged 40–79 years, were invited to take part, and 6004 participated (48.2%), whereof 3872 persons had previously participated in SAMINOR 1. The present analyses are based on longitudinal data including individuals participating in both SAMINOR 1 and SAMINOR 2 who did not fill the exclusion criteria detailed below.

We lack information about those invited to SAMINOR 2, who had also participated in SAMINOR 1 but who failed to participate in SAMINOR 2, as a linkage is only allowed for those who participated in both surveys. Therefore, loss to follow-up is described based on SAMINOR 1 participants who would have been invited to SAMINOR 2, given that they had not died or moved from the 10 studied municipalities prior to invitation to SAMINOR 2. There were 11,558 invitees to SAMINOR 1, who, according to their birth year and municipality, would have been invited to SAMINOR 2, given that they had not moved or died. Of these, 6450 (55.8%) participated in the SAMINOR 1 clinical examinations, of whom 6408 gave their consent to register linkages. The two data files were merged by Statistics Norway, using the unique 11-digit personal identification number assigned to all subjects residing in Norway.

**Fig. 2:**
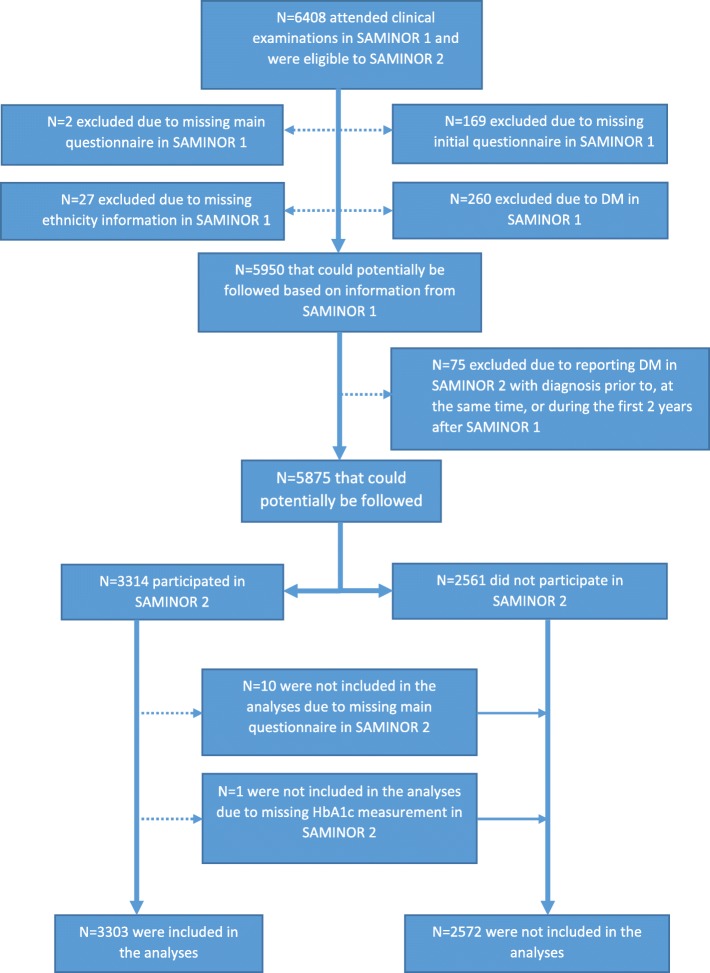
Flow chart demonstrating persons included for final analysis

Figure 2 displays the population and exclusions applied. Among the 6408 individuals, the following were excluded: 169 due to missing initial questionnaire; 2 due to missing main questionnaire (containing diabetes information); and 27 due to missing ethnicity information in SAMINOR 1. Based on self-report and random (non-fasting) plasma glucose (RPG) ≥11.1 mmol/L measurement in SAMINOR 1, 260 prevalent cases of DM were excluded. To ensure exclusion of prevalent cases, in total 75 participants were excluded, as, in SAMINOR 2, they reported the date at the time of DM diagnosis as prior to (*n* = 52), at the same time as (*n* = 6) or during the first 2 years after participating in SAMINOR 1 (*n* = 17, 2 years wash-out period). Of the remaining 5875 persons, 11 were not included in the final analysis due to missing main questionnaire (*n* = 10) or HbA1c measurement (*n* = 1) in SAMINOR 2. A total of 2561 did not participate in SAMINOR 2 as they had died, moved out of the included municipalities during the follow-up period, or were not willing or able to participate in SAMINOR 2. Hence, 3303 individuals (follow-up rate: 56.2%) were included in the analysis (Fig. 2).

The data collection for SAMINOR 1 took place over two calendar years and over three calendar years for SAMINOR 2, and the municipalities were not visited in the same order in the two surveys. Thus, the time span between the two examinations varied from 8 to 11 years, with a mean of 10.1 years. The merged file contains individuals born in the period 1933–1968 and in 1973, who were aged 30 and 36–71 years in SAMINOR 1 and 40–41 and 44–79 years in SAMINOR 2.

### Blood sampling

In both SAMINOR 1 and 2, blood samples were taken by venipuncture at normal venous pressure with the participant in a seated position. In SAMINOR 1, blood samples were mailed directly to the laboratory for analysis. Among the included analyses was RPG. The applied methods and procedures in SAMINOR 1 are described in detail elsewhere [12]. In SAMINOR 2, glycated haemoglobin (HbA1c) was measured immediately on site from whole blood, using DCA Vantage™ (Siemens Medical Solutions Diagnostics, Tarrytown, NY, USA). In SAMINOR 1, HbA1c was not measured.

### Ethnicity

Ethnic information was collected through self-report in SAMINOR 1. The questions were: “What language(s) do/did you, your parents and your grandparents use at home?”, “What is your, your father’s and your mother’s ethnic background?”, and “What do you consider yourself to be?” For all items, the response options were: “Norwegian”, “Sami”, “Kven”, and “Other”. The questions were to be answered separately for each relative, and multiple answers were allowed. Sami ethnicity was defined based on two criteria: 1) self-identification as a Sami, and 2) a Sami language connection. Sami self-identification was regarded as fulfilled if the respondent considered him/herself to be Sami or reported having a Sami ethnic background. Sami language connection was defined if at least one grandparent, parent, or the participant him/herself spoke a Sami language at home. Participants who fulfilled both criteria were categorised as Sami. All other participants were categorised as non-Sami.

### Diabetes mellitus

In SAMINOR 1, both questionnaire information and RPG levels were used to categorized participants as having DM. The question concerning diabetes was: “Do you have, or have you had, diabetes? (yes/no)”. Those who answered “yes”, or who had RPG levels of 11.1 mmol/L or higher, were considered prevalent cases of DM.

In SAMINOR 2, the question was: “Have you ever been diagnosed with diabetes (elevated blood sugar levels)? (yes/no)”. Missing self-report of DM was classified as “no”. Participants who answered “yes” or had HbA1c ≥ 6.5% were categorised as incident cases.

### Risk factors for type 2 DM

All potential risk factors for DM included in the present study were measured at the start of the study, i.e., in SAMINOR 1.

Height (cm) and weight (kg) were measured using an electronic height and weight scale, with participants wearing light clothing without shoes. Body mass index (BMI) was calculated as weight in kilograms, divided by the square of the height in metres (kg/m^2^). Waist circumference (WC) was measured in centimetres at the umbilicus, with the participant standing and breathing normally. Waist-to-height ratio (WHtR) was calculated as waist circumference divided by height.

Those who reported in the questionnaire that at least one of their parents, siblings or offspring had DM were regarded as having a positive family history of DM. Marital status (married vs single, widowed/widower, divorced or separated), education (highly educated with more than 12 years of education vs lower education), cigarette smoking (current smoker vs ex-smoker or never-smoker), alcohol drinking (drinking at least once a week vs drinking less often), annual family gross income (lower than 451,000 Norwegian Kroner vs higher income) were also assessed.

Hopkins Symptom Checklist (SCL-10) was used for measuring mental distress [15]. Ten items relevant for mental health are included in the SCL-10: experiencing fear, frightened/ anxiousness, faintness/dizziness, tenseness/upset, insomnia/sleeplessness, easily blaming yourself, being dejected/melancholia, being useless or of little value, experiencing everything as a struggle, being hopeless regarding the future. Each question was answered on a four-point scale ranging from 1 = “Not affected” to 4 = “Extremely affected”. In total, 418 participants had at least one missing answer to one of the mentioned ten questions. Imputation was performed for those with one (*n* = 130) or two (*n* = 31) missing answers, by assigning the mean values of the respective questions to them, as described by Strand et al. [16]. For records with three or more missing responses, the SCL-10 score was not calculated. The mean of the ten scores was then calculated for each participant, by dividing the sum of the scores by ten. A SCL-10 score over 1.85 is considered indicative of mental distress [15, 16].

Participants scored their leisure-time physical activity during the past year on a four-point scale: 1) “reading, watching TV, or other sedentary activities”; 2) “walking, cycling, or similar forms of exercise at least 4 h a week”; 3) “at least 4 h a week of recreational sports, heavy gardening, etc.”; and 4) “hard training or sports competitions regularly and several times a week” [17]. Those who reported reading, watching TV, or other sedentary activities were regarded as inactive.

### Statistical analysis

Data management and statistical analysis were performed using Stata version 15.0 (Stata Corp., College Station, TX, USA). All tests were two-sided with a 5% significance level.

Those who were included in the analysis were compared with those we would wish to follow up but were not able to include (due to death, emigration, or lack of participation or insufficient information in SAMINOR 2) with regard to the available baseline characteristics and risk factors for DM (Table 1). Differences in mean age, BMI, WC, and WHtR were tested by two-sample *t*-tests. For the categorical variables, Sami ethnicity, having positive family history of DM, marital status, being highly educated, SCL-10 score > 1.85 (mental distress), smoking, drinking alcohol, having low income, and being inactive in leisure-time, the groups were compared using Pearson’s χ^2^ tests. The same variables were compared for Sami vs non-Sami subjects included in the analyses (Table 2).

**Table 1:** Characteristics of individuals we were able to follow-up, compared to those who were not followed up, among those who participated in SAMINOR 1 (2003–2004) and were eligible^a^ for SAMINOR 2 (2012–2014), by sex (*N* = 5875). Numbers are mean (standard deviation) for continuous variables and percent (number of subjects) for categorical variables

	Included in the follow-up analysis	Not included in the follow-up analysis	*p*-value
Men	*N* = 1447	*N* = 1307	
Age (year)	52.4 (8.7)	51.2 (9.8)	< 0.01
Body mass index (kg/m^2^)	27.5 (3.5)	27.6 (4.2)	0.42
Waist circumference (cm)	92.3 (9.3)	93.0 (10.9)	0.07
Waist-to-height ratio	0.534 (0.054)	0.537 (0.064)	0.10
Sami ethnicity (%)	40.2 (581)	32.7 (866)	< 0.01
Family history of DM^b^ (%)	19.4 (280)	18.2 (238)	0.44
Married^c^ (%)	64.5 (933)	52.8 (690)	< 0.01
Education> 12 years (%)	32.8 (458)	30.7 (381)	0.26
SCL-10 score > 1.85 (%)	5.3 (72)	9.5 (114)	< 0.01
Current smoker^d^ (%)	28.8 (416)	39.5 (516)	< 0.01
Alcohol^e^ (%)	30.7 (444)	31.1 (407)	0.80
Low-income^f^ (%)	57.0 (825)	61.5 (804)	0.02
Inactive^g^ (%)	18.8 (272)	23.1 (302)	0.01
Women	*N* = 1856	*N* = 1265	
Age (year)	51.6 (9.0)	50.7 (10.1)	< 0.01
Body mass index (kg/m^2^)	27.4 (4.6)	27.6 (4.9)	0.38
Waist circumference (cm)	84.0 (11.2)	84.2 (11.8)	0.08
Waist-to-height ratio	0.526 (0.074)	0.527 (0.076)	0.40
Sami ethnicity (%)	39.5 (733)	29.4 (372)	< 0.01
Family history of DM^b^ (%)	23.2 (430)	21.8 (276)	0.38
Married^c^ (%)	66.0 (1225)	58.2 (736)	< 0.01
Education> 12 years (%)	38.0 (674)	36.3 (428)	0.34
SCL-10 score > 1.85 (%)	8.4 (141)	11.5 (130)	< 0.01
Current smoker^d^ (%)	30.6 (568)	40.9 (517)	< 0.01
Alcohol^e^ (%)	19.7 (365)	20.5 (259)	0.58
Low-income^f^ (%)	58.7 (1090)	62.7 (793)	0.03
Inactive^g^ (%)	19.1 (355)	22.9 (289)	0.01

^a^Living in the 10 SAMINOR 2 municipalities at time of SAMINOR 1 with relevant year of birth

^b^Those who had at least one with DM among father, mother, siblings or children

^c^Married vs single, widow/widower, divorced, or separated

^d^Current smokers vs former smokers or never-smokers

^e^Drinking alcohol at least once a week

^f^Yearly gross income of the household less than 451,000 Norwegian Kroner

^g^Leisure-time activities include reading, watching TV or other sedentary activities

**Table 2:** Baseline characteristics of diabetes-free participants in SAMINOR 1 (2003–2004) followed-up to SAMINOR 2 (2012–2014), *N* = 3303. Numbers are mean (standard deviation) for continuous variables (age, body mass index, waist circumference, and waist-to-height ratio) and percent (number of subjects) for categorical variables (family history of DM, married, education> 12 years, SCL-10 score > 1.85, alcohol, low-income, and inactive)

	Sami	Non-Sami	*p*-value
Men	*N* = 581	*N* = 866	
Age (year)	51.8 (8.8)	52.8 (8.7)	0.04
Body mass index (kg/m^2^)	27.8 (3.8)	27.3 (3.3)	0.02
Waist circumference (cm)	91.7 (9.8)	92.8 (9.0)	0.03
Waist-to-height ratio	0.540 (0.060)	0.529 (0.050)	< 0.01
Family history of DM^a^ (%)	20.5 (119)	18.6 (161)	0.37
Married^b^ (%)	59.2 (344)	68.0 (589)	< 0.01
Education> 12 years (%)	32.6 (184)	32.9 (274)	0.89
SCL-10 score > 1.85 (%)	6.3 (34)	4.6 (38)	0.17
Current smoker^c^ (%)	29.6 (172)	28.2 (244)	0.55
Alcohol^d^ (%)	27.4 (159)	32.9 (285)	0.02
Low-income^e^ (%)	60.2 (350)	54.8 (475)	0.04
Inactive^f^ (%)	20.3 (118)	17.8 (154)	0.23
Women	*N* = 733	*N* = 1123	
Age (year)	50.7 (8.9)	52.1 (8.9)	< 0.01
Body mass index (kg/m^2^)	28.0 (4.8)	27.0 (4.5)	< 0.01
Waist circumference (cm)	84.5 (11.3)	83.6 (11.2)	0.11
Waist-to-height ratio	0.539 (0.075)	0.516 (0.072)	< 0.01
Family history of DM^a^ (%)	24.6 (180)	22.3 (250)	0.25
Married^b^ (%)	60.3 (442)	69.7 (783)	< 0.01
Education> 12 years (%)	42.7 (298)	35.0 (376)	< 0.01
SCL-10 score > 1.85 (%)	9.0 (60)	8.0 (81)	0.47
Current smoker^c^ (%)	31.6 (232)	29.9 (336)	0.43
Alcohol^d^ (%)	14.3 (105)	23.1 (260)	< 0.01
Low-income^e^ (%)	61.0 (447)	57.3 (643)	0.11
Inactive^f^ (%)	25.0 (183)	15.3 (172)	< 0.01

^a^Those who had at least one with DM among father, mother, siblings or children

^b^Married vs single, widow/widower, divorced, or separated

^c^Current smokers vs former smokers or never-smokers

^d^Drinking alcohol at least once a week

^e^Yearly gross income of the household less than 451,000 Norwegian Kroner

^f^Leisure-time activities include reading, watching TV or other sedentary activities

Those who were categorised as having DM in SAMINOR 2, but not in SAMINOR 1 or the first 2 years after it, were regarded as incident cases of DM, and, by dividing the number of incident cases by the number of DM-free participants in SAMINOR 1 (at-risk individuals), the approximate 8-year cumulative incidence of DM was estimated.

Multiple logistic regression analysis was used to assess the effect of ethnicity (Sami vs non-Sami), as well as various risk factors, on the development of DM (Table 3). At first, the effect of each potential risk factor was assessed using univariable regression analyses. Then the variables with significant ORs in the univariable analyses were included together with ethnicity and sex in a multivariable logistic regression analysis. Of BMI, WC and WHtR only WHtR was put into the multivariable analysis as the three variables have a large correlation with each other. Family history of DM was not put in the multivariable analysis to avoid over-adjustment (Table 3).

**Table 3:** Univariable and multivariable odds ratios (OR) with 95% confidence interval (95% CI) for incident cases of diabetes mellitus (DM) for various possible risk factors for DM. SAMINOR 1 (2003–2004) and SAMINOR 2 (2012–2014), *N* = 3303

Models	OR (95% CI)	*p*-value
Sami ethnicity	1.01 (0.75–1.34)	0.96
age	1.03 (1.01–1.05)	< 0.01
sex (female)	0.83 (0.62–1.01)	0.19
BMI^a^	1.18 (1.15–1.22)	< 0.01
WC^b^	1.07 (1.06–1.08)	< 0.01
WHtR^c^	1.12 (1.10–1.15)	< 0.01
education (year)	0.92 (0.88–0.96)	< 0.01
inactivity^d^	1.32 (0.94–1.86)	0.10
alcohol^e^	0.73 (0.51–1.04)	0.08
smoking^f^	0.98 (0.71–1.33)	0.89
mental distress^g^	0.69 (0.39–1.37)	0.29
family history of DM^h^	2.77 (2.07–3.73)	< 0.01
ethnicity+age + sex+ WHtR+education	Sami: 0.81 (0.59–1.11)	0.20
	age: 1.01 (0.99–1.02)	0.46
	sex (female): 0.71 (0.52–0.97)	0.03
	WHtR: 1.13 (1.10–1.15)	< 0.01
	education: 0.96 (0.92–1.01)	0.12

^a^BMI: body mass index (kg/m^2^)

^b^WC: waist circumference (cm)

^c^WHtR: waist-to-height ratio. In order to help understanding, this variable is multiplied by 100

^d^Leisure-time physical activity includes reading, watching TV or other sedentary activities

^e^Drinking alcohol at least once a week vs drinking alcohol less often

^f^Current smokers vs ex-smokers and never-smokers

^g^SCL-10 score > 1.85

^h^Those who had at least one with DM among father, mother, siblings or children

### Ethics

The SAMINOR Study was approved by the Norwegian Data Inspectorate and by the Regional Committees for Medical and Health Research Ethics North (REC North). The committee also approved the present study, with approval number 2016/173. All participants gave written informed consent for medical research and to have their data linked to other registers or surveys. The study was also approved by the SAMINOR Project Board.

## Results

Compared to subjects who took part in SAMINOR 1, but were not followed up, subjects who participated in both surveys were on average older, and more likely to be married and report Sami ethnicity. Furthermore, those included in the follow-up analyses were more physically active, and less likely to be current smokers, reporting mental disorders and having low income (Table 1).

Table 2 shows some baseline characteristics of DM-free individuals in SAMINOR 1 who were followed up until SAMINOR 2. In both sexes, Sami had higher mean WHtR and BMI compared to non-Sami. Mean WC was higher among non-Sami men, while no statistically significant difference was observed in the mean WC between Sami and non-Sami women. Among women, more Sami than non-Sami were considered inactive (Table 2).

A total of 201 incident cases of DM were identified in SAMINOR 2, based on self-report (*n* = 138) or HbA1c ≥ 6.5% (without self-report) (*n* = 63). We noted that all the self-reported cases had HbA1c ≥ 6.5% (results not shown). This number corresponds to a 6.1% (95% confidence interval: 5.3–6.9) 8-year cumulative incidence of DM. The 8-year cumulative incidence of diabetes among Sami and non-Sami men was 7.1% (95% confidence interval: 5.1–9.5) and 6.5% (95% confidence interval: 4.9–8.3) respectively. Corresponding values for Sami and non-Sami women were 5.3% (95% confidence interval: 3.8–7.2) and 5.8% (95% confidence interval: 4.5–7.3%) respectively.

In univariable analyses, higher age, BMI, WC, and WHtR, lower education and having positive family history of DM significantly increased the odds for incident DM (Table 3). Women had statistically significantly lower incidence of DM when adjusting for ethnicity, age, WHtR and education. No statistically significant difference was found between Sami and non-Sami in the odds of 8-year cumulative incidence of DM.

## Discussion

The present study is the first to estimate the cumulative incidence of DM among Sami and non-Sami inhabitants of Northern Norway. After 8 years of follow-up, 201 (6.1%) incident cases of DM were identified, based on self-report and/or HbA1c ≥ 6.5%. The 8-year cumulative incidence of DM was not statistically significantly different between Sami and non-Sami.

Of 5875 SAMINOR 1 participants who were eligible to participate in SAMINOR 2, 3303 were included in the follow-up analysis. To assess the risk of selection bias, we compared some relevant and available risk factors for DM between those who were included in the analysis and those who were not. Although those who were not included in the final analysis were on average younger, the age discrepancy was only around 1 year, which may not have affected the estimated cumulative incidence of DM. Not being married, being a smoker, having a higher SCL-10 score (mental distress indicator), having lower income and having lower level of leisure-time physical activity, were some attributes of those who were not included in the analysis. In the second survey of the Tromsø Study, it was found that non-participants were over-represented among young and unmarried men [18]. Results from the Tromsø Study indicate lower mortality in subjects who attended several surveys rather than only one [19]. Results from similar studies in Norway indicate that non-participants have higher levels of chronic diseases and higher mortality rates; furthermore, non-participants are more likely to be receiving disability pension and belonging to lower socioeconomic groups [20, 21]. On the other hand, BMI, WC, WHtR (indicators of obesity) and having a positive family history of DM (an indicator of genetic predisposition to DM) were not statistically significantly different between those included in our analysis and those not, making it less likely that the two groups were systematically different with regard to the risk of DM.

If loss to follow-up is due to the outcome (DM), its complications or diseases with shared risk factors (e.g. cardiovascular diseases), the cumulative risk is underestimated (competing risk effect) [22]. Our dataset was not linked to the Cause of Death Registry, so we do not have direct information about the number and causes of death of those who died during the follow-up period. It is unlikely that a participant who contracted DM during the follow-up period and died of the disease itself or its late complications. On the other hand, deaths due to competing risks (like cardiovascular diseases) inevitably lead to underestimation of the cumulative incidence of DM. Based on numbers from Statistics Norway, one can expect there to have been around 330 deaths from 2001 to 2011 (10 years) in a group of 5875 persons with similar age distribution to those of our participants (calculations not shown) [23].

According to the Norwegian Institute of Public Health, cancers are the leading cause of death in people with a similar age span to those of our participants, followed by cardiovascular diseases (mutual risk factors for DM) [24]. Competing risks become more important with the increasing age of the population under study (increased risk of multimorbidity). As the mean baseline age of both groups, those that were followed up and those that were not, was around 52 years, and there were relatively few expected deaths (330 deaths totally), it is not thought that competing risks have substantially affected our estimate of the cumulative incidence of DM. Furthermore, studies have shown minimal or no difference between Sami and non-Sami individuals in the distribution of risk factors for cardiovascular diseases and/or the risk of acute myocardial infarction or cerebral stroke [25, 26]. We do not have information on the participants in SAMINOR 1, who, due to emigration, were not included in the final analysis, but they were few, and it is unlikely that they had any impact on the conclusions.

At the end of the follow-up period (SAMINOR 2), self-reported DM and/or HbA1c ≥ 6.5% was used to identify incident cases of DM. This HbA1c cut-off is recommended by the American Diabetes Association, as well as the Norwegian Directorate of Health [27, 28], and is being largely applied in clinical practice. According to the Tromsø OGTT study, an HbA1c cut-off ≥6.5% provides sensitivity and specificity of around 35 and 97%, respectively [29]. The low performance of the test leads to substantial misclassification of DM, but it must be assumed to be unrelated to categorization as a Sami or not.

The questionnaire applied in the present study was not validated. However, the sensitivity and positive predictive value of self-reported DM were reported as 86.7 and 73.4%, respectively, in the CADEUS study in France, using medical records as standard [30]. The validity of self-reported DM in the HUNT 1 Survey was reported to be excellent by comparison with the general practitioners’ records, with positive and negative predictive values of 96 and 99.7%, respectively [31].

The lack of statistically significant difference in the 8-year cumulative incidence of DM between Sami and non-Sami might be explained by the misclassifications or the relatively small study sample size. Similar standards of living, high awareness about lifestyle diseases like type 2 DM and fair access to healthcare services for both ethnic groups in the study municipalities, are other possible explanations.

According to a recently published cohort study, the estimated prevalence of diagnosed type 2 DM for all residents in Norway aged 30 to 89 years increased from 4.9% in year 2009 to 6.1% in 2014 [32]. Nevertheless, the incidence rate of type 2 DM decreased significantly from 609 cases per 100,000 person-years in 2009 to 398 cases per 100,000 in 2014, an annual reduction of 10.1%. Our estimated cumulative incidence of DM (6.1% in 8 years or around 762 cases in 100,000 participants in a year) is comparable to the reported 609 cases per 100,000 person-years in year 2009. It should be kept in mind that our estimate included all types of DM, while the mentioned study reported known cases of type 2 DM only. However, due to the age of the new cases, they must be expected to be mainly type 2 DM. In the HUNT Study (from 1995 to 1997 to 2006–2008), the 11-year cumulative incidence of any diabetes was around 4.5% among adults (20 ≤ age < 70) using self-report, RPG ≥ 11.1 mmol/L, fasting plasma glucose ≥7 mmol/L, HbAc1 ≥ 6.5% or 2-h 75 g OGTT ≥11.1 mmol/L [31]. The different age span of participants in the HUNT Study is the most likely explanation for the difference between our results and those from the HUNT Study.

Results from the present study, as well as results from our previous studies, which found either no or not a marked ethnic difference in the incidence or prevalence of DM between Sami and non-Sami people in Norway [8–10], imply substantial better conditions for Sami people in Norway, compared with those of other indigenous peoples throughout the world. This is probably due to the Sami enjoying quite similar living and healthcare standards to those of other Norwegian citizens.

### Strengths and limitations

Some of the strengths of the present study lie in the application of a comprehensive questionnaire and the use of trained personnel, enabling us to obtain copious amounts of information on several aspects of living and health-related conditions, as well as the use of HbA1c, in addition to self-report, to ascertain DM. The present study is the first longitudinal study to measure the cumulative incidence of DM in Sami-inhabited regions in Norway.

A suboptimal participation rate, relatively small sample size, limited number of included municipalities, non-fasting glucose measurements, lack of sufficient dietary information, no differentiation between types of DM, lack of linkage to national health registers such as prescription databases, the Cause of Death Register, or discharge register, are limitations of the present study. As a large number of people were included, confirmation of diabetes diagnosis with 2-h post-prandial glucose measurement was not feasible. It is also a limitation that we lack information about which of the SAMINOR 1 participants were actually invited to SAMINOR 2.

We did not have reliable data on the exact time of diagnosis/occurrence of the disease, which made calculation of the incidence rate of DM impossible.

## Conclusions

We observed no ethnic difference in the 8-year cumulative incidence of DM, although mean WHtR and BMI were higher among Sami than non-Sami participants of both sexes. There may be a need for larger studies in the future, to track and elucidate any ethnic difference in the cumulative incidence or incidence rate of DM.

## Data Availability

The data that support the findings of this study were used under license for the current study and are therefore not publicly available. Data are available from the SAMINOR Study upon reasonable request (www.saminor.no), but restrictions apply to the availability of these data, due to Norwegian privacy regulations.

## Abbreviations

BMI: Body mass index
DM: Diabetes mellitus
HbA1c: Glycated hemoglobin
RPG: Random plasma glucose
TV: Television
WC: Waist circumference
WHtR: Waist-to-height ratio
